# Observed shifts in the contact zone between two forms of the diving beetle *Hydroporus memnonius* are consistent with predictions from sexual conflict

**DOI:** 10.7717/peerj.2089

**Published:** 2016-06-14

**Authors:** David T. Bilton, Garth N. Foster

**Affiliations:** 1 Marine Biology & Ecology Research Centre, University of Plymouth,United Kingdom; 2 Aquatic Coleoptera Conservation Trust,Ayr,Scotland,United Kingdom

**Keywords:** Sexual conflict, Intrasexual dimorphism, Population, Dytiscidae, Biogeography

## Abstract

Sexual conflict drives both inter- and intrasexual dimorphisms in many diving beetles, where male persistence and female resistance traits co-evolve in an antagonistic manner. To date most studies have focussed on species where rough and smooth females and their associated males typically co-occur within populations, where phenotype matching between morphs may maintain forms as stable polymorphisms. The Palaearctic diving beetle *Hydroporus memnonius* is characterised by having dimorphic (rough var. *castaneus* and smooth, shining) females and associated males which differ in persistence traits; the two forms being largely distributed parapatrically. In this species, instead of mating trade-offs between morphs, males associated with *castaneus* females should have a mating advantage with both this form and shining females, due to their increased persistence abilities on either cuticular surface. This may be expected to lead to the replacement of the shining form with *castaneus* in areas where the two come into contact. Using data collected over a thirty year period, we show that this process of population replacement is indeed occurring, *castaneus* having expanded significantly at the expense of the shining female form. Whilst populations of both forms close to the contact zone appear to differ in their thermal physiology, these differences are minor and suggest that the expansion of *castaneus* is not linked to climatic warming in recent decades. Instead we argue that the observed spread of *castaneus* and its associated male may result from the dynamics of sexually antagonistic coevolution in this beetle.

## Introduction

Sexual conflict can drive the evolution of reciprocal adaptations and counter-adaptations in males and females, including pronounced morphological differences between the sexes (Chapman et al., 2003; Parker, 2006). Such sexual dimorphism is frequent in diving beetles (Dytiscidae--- Sharp, 1882; Balfour-Browne, 1940, Balfour-Browne, 1950; Bergsten & Miller, 2007; Bilton, Thompson & Foster, 2008), where males often possess modified, sucker-like articulo-setae on the tarsi of their fore- and middle legs which increase their ability to grasp females during mating (Aiken, 1992; Aiken & Khan, 1992). In contrast, females often have marked modifications to their dorsal sculpture, which make it harder for males to grasp females during what may be aggressive and prolonged pairings (Régimbart, 1877; Blunck, 1912; Wesenberg-Lund, 1912; Smith, 1973; Aiken, 1992). Mating may increase female vulnerability to predation, as well as incurring energetic costs. Additionally, in many species it seems that males actively restrict female access to surface air during pairing, behaviour likely to incur metabolic costs (Miller & Bergsten, 2014). The phylogenetic distribution of sexually dimorphic traits, which are scattered across the Dytiscidae, suggest that they may have evolved multiple times in response to the escalation of sexual arms races (Miller, 2003; Miller & Bergsten, 2014).

Pronounced intrasexual dimorphism is also seen in some species of diving beetle (Bilton, Thompson & Foster, 2008), where two distinct female forms are present; one male-like, the other with modified surface sculpture, these forms sometimes being associated with morphologically distinct males. In a few cases there is a strong geographical component to this variation, modified females being present at higher frequency in some part of the species' range, or almost complete allo/parapatry occurring between the two forms (Balfour-Browne, 1940, Balfour-Browne, 1950; Foster, Bilton & Nelson, 2016), although the drivers of such patterns remain unclear (e.g., Sharp, 1882; Roughley & Pengelly, 1981). One of the most striking cases of this phenomenon is seen in *Hydroporus memnonius* Nicolai, a widespread west Palaearctic species (Balfour-Browne, 1940; Zaitsev, 1972; Drost et al., 1992; Nilsson & Holmen, 1995; National Biodiversity Network Gateway, 2016).

**Figure 1:**
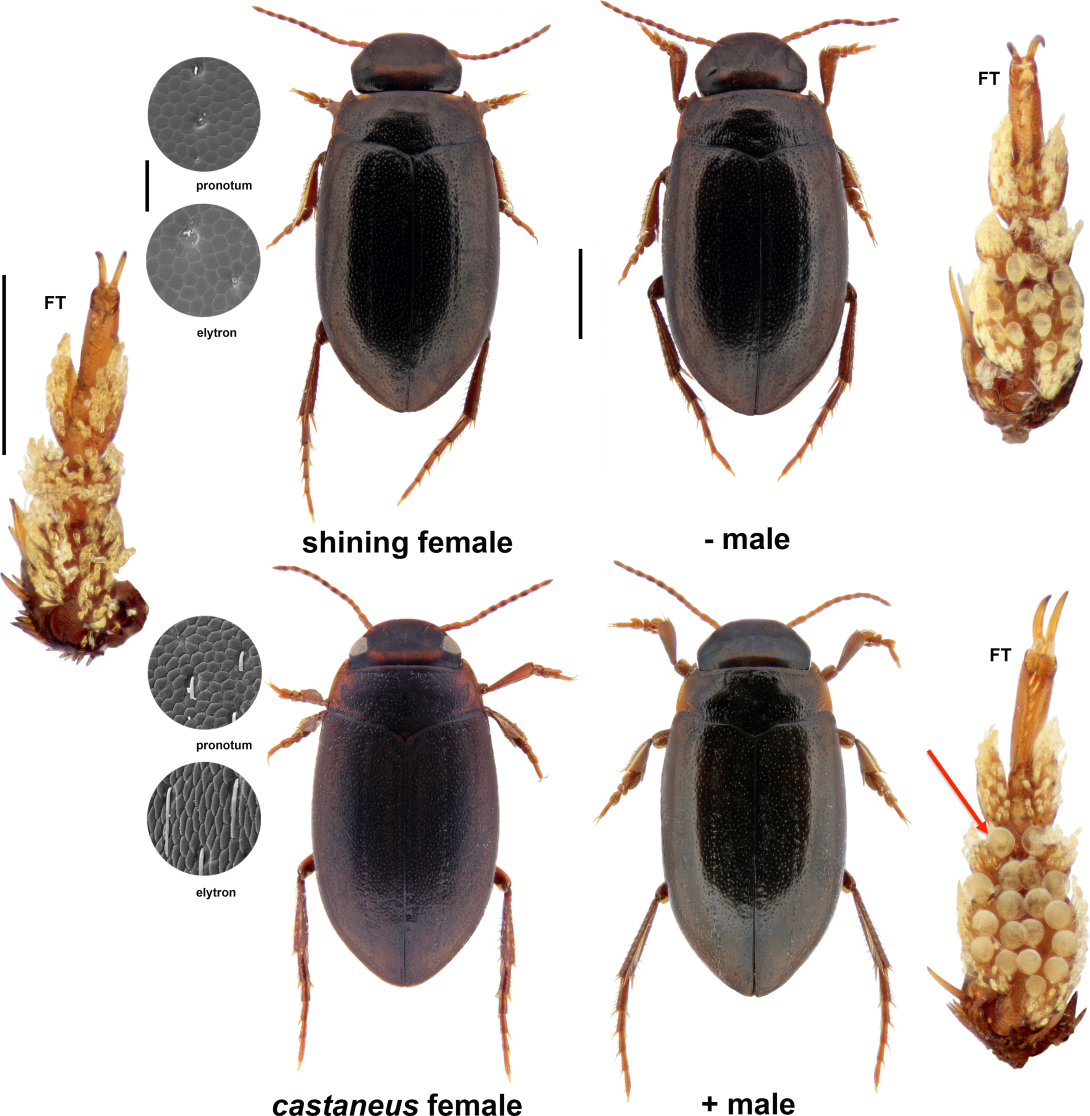
Morphological features of *Hydroporus memnonius* morphs. Shown are habitus images of female and male morphs, together with scanning electron micrographs of female pronotal and elytral microsculpture, and images of the ventral sides of foretarsi (FT). Male microsculpture in both forms is identical to that of shining females; mid-tarsal anatomy essential equivalent to that of foretarsi. Red arrow on + male foretarus indicates one of the pair of large adhesive suckers on the second tarsal segment, characteristic of this morph and absent from − males (see text). Scale bars as follows: whole beetles 1 mm; foretarsi 250 µm; microsculpture 50 µm.

As discussed by Bilton, Thompson & Foster (2008) *H. memnonius* females are strikingly dimorphic, having either smooth, shining, male-like dorsal surfaces which are weakly reticulate, or denser, more intense surface microsculpture, which gives them a rougher, matt appearance, even to the naked eye (e.g., Balfour-Browne, 1940--- see Fig. 1). The matt female form is indeed so distinct that it has attracted the name *castaneus* Aubé, in reference to the reddish colouration sometimes associated with the form. The distribution of the two female forms, which do not appear to differ in ecology, has attracted the attention of earlier workers (e.g., Fairmaire & Laboulbène, 1854; DesGozis, 1910--1915; Guignot, 1930--1933), and whilst there are regions where the two co-occur, these are limited. Matt females are known from northern and central Europe, as well as Italy and the northern Balkans (e.g., Franciscolo, 1979), but are largely absent from many areas of the species' range including Scandinavia and Iberia. Shining female populations are known from Britain and Ireland, France, Italy, Iberia, Greece and Scandinavia. The distribution of *castaneus* and shining females is best documented in the British Isles, where the two forms are almost entirely allopatric (Balfour-Browne, 1940; Bilton, Thompson & Foster, 2008; Foster, Bilton & Nelson, 2016). Shining female populations are the only form of *H. memnonius* known from Ireland, the Isle of Man, Anglesey and almost all of Scotland, and are also present to the exclusion of the matt form in some parts of the Lake District, west Wales and the Isles of Scilly (Bilton, Thompson & Foster, 2008; Foster, Bilton & Nelson, 2016). Only matt females are known from most of England, with a contact zone between the two forms in Scotland immediately north of the Border with England, and in parts of west Wales and Cumbria.

Bilton, Thompson & Foster (2008) demonstrated that the two forms of female are associated with morphologically distinct males; those from *castaneus* populations having a higher number of sucker-like articulo-setae on their tarsi, some of which are individually larger, as well as a different distribution of suckers across tarsomeres of both front and middle legs (see Fig. 1). Males associated with *castaneus* females (hereafter referred to as + males) have a pair of large adhesive suckers on the second tarsal segment of both fore and middle legs, which are consistently absent in males from shining female populations (hereafter referred to as − males). Despite these differences, in both females and males, mitochondrial DNA sequence data show that the two forms do not represent reciprocally monophyletic clades (see Bilton, Thompson & Foster, 2008). The larger, more abundant tarsal suckers of + males have been interpreted as a male counter-measure, evolved in response to the increased female surface sculpture in this form, since an increase in sucker number and size will increase both attachment time and suction force (see Bergsten & Miller, 2007 for details of the physics of attachment devices). In the case of *H. memnonius*, as well as aiding their ability to grasp *castaneus* females, the tarsal characteristics of + males should significantly enhance an individual's ability to mate with shining, male-like females, since larger suction cups are also more effective on a smooth surface (Aiken & Khan, 1992; Bergsten & Miller, 2007).

Bilton, Thompson & Foster (2008) suggested that the contact zone between *castaneus* and shining females is likely to be dynamic, and that the interaction may be governed, at least in part, by the relative mating success of the two male forms where populations meet. In particular, the greater predicted persistence ability of + males may drive their expansion at the expense of the − form, with the *castaneus* female increasing as a result of higher fitness associated with lower mating frequency, and/or linkage disequilibrium between male and female traits. Such a scenario predicts that *castaneus* will gradually replace the shining female in areas where the two meet, an idea which is explored explicitly in the present paper.

Here we report on the composition of *Hydroporus memnonius* populations spanning the contact zone between *castaneus* and shining females in northern England and southern Scotland, examining whether *castaneus* females and associated + males are expanding their range, by comparing data from the present survey with historical records from the 1970s and 1980s, when the contact zone's position was first identified. Using samples from all known mixed populations we also explore whether the frequency of + males is positively related to that of *castaneus* females. Viewed globally, the comparative distribution of *castaneus* and shining female populations (see above) is not readily predicted by differences in climate (mediated through differences in thermal physiology---see Calosi et al., 2010), since shining females appear to be largely a feature of peripheral areas of the species' range, both north and south. Despite this, the time period involved in our comparison of the contact zone in the UK has seen a shift in climate which has driven northward range expansions in a number of European invertebrates (e.g., Hickling et al., 2006; Thomas, 2010; Lenoir & Svenning, 2015). Long-term temperature records show that Scottish climate has warmed over the time period, the smoothed kernal filter values of mean daily maximum and minimum temperatures increasing by approx. 0.8 and 0.6 °C respectively, with all years since 2000 having mean annual daily maxima at least 0.5 °C warmer than the 1961--1990 average in the study area (UK Met Office, 2016). In light of these changes, we explore the comparative thermal physiology of *castaneus* and shining female populations from either side of the UK contact zone, in an attempt to determine whether any observed changes in distribution reflect the relative temperature tolerance of the two forms.

**Table 1:** Populations of *Hydroporus memnonius* sampled, with past and present statuses, and percentage of *castaneus* females and associated + males in 2007--2008.

Locality	UK Grid Ref	Past status (year)	Status 2007--08	*N* ♀	% *castaneus* ♀	*N* ♂	% + ♂
England Cumberland Moorthwaite Moss	NY51-50-	Matt (1983)	Matt	21	100	24	100
England Cumberland Tarn Moss	NY39-27-	Matt (1980)	Matt	21	100	18	100
England Cumberland White Moss	NY45-60-	Matt (1985)	Matt	31	100	38	100
England Cumberland Solway Moss	NY35-70-	Matt (1981)	Matt	38	100	21	100
England Cumberland Gelt Woods	NY52-58-	Matt (1983)	Matt	24	100	17	100
Scotland Dumfriesshire Archer Beck	NY41-79-	Matt (1982)	Matt	23	100	23	100
Scotland Berwickshire Lurgie Loch Moss	NT67-39-	Matt (1985)	Matt	32	100	40	100
Scotland Berwickshire Gordon Moss	NT63-42-	Matt (1976)	Matt	12	100	20	100
Scotland Selkirkshire Riskinhope Moss	NT23-19-	Matt (1985)	Mixed	21	95.2	33	100
Scotland RoxboroughTandlaw Moss	NT48-17-	Mixed (1975)	Matt	9	100	26	100
Scotland Roxborough Blackpool Moss	NT51-29-	Mixed (1974)	Matt	21	100	17	100
Scotland Roxborough Dunhog Moss	NT47-24-	Mixed (1974)	Mixed	10	30	26	42.3
Scotland Roxborough Harden Moss	NT44-16-	Mixed (1975)	Mixed	13	7.7	7	14.3
Scotland Roxborough Whitmuir Hall	NT50-27-	Mixed (1974)	Mixed	36	69.4	21	76.2
Scotland East Lothian Coulstoun Wood	NT53-70-	Mixed (1984)	Mixed	12	0	26	7.7
England Cumberland Hangingshaw Moss	NY11-46-	Shiny (1985)	Mixed	29	24.1	44	22.7
England Cumberland Beckgrain Bridge	NY19-35-	Shiny (1987)	Mixed	15	13.3	17	11.8
England Cumberland Dubbs Moss	NY10-28-	Shiny (1987)	Mixed	25	8.0	7	0
England Cumberland Mockerkin Tarn	NY08-23-	Shiny (1987)	Mixed	19	5.3	19	0
Scotland Dumfriesshire Caerlaverlock	NY04-64-	Shiny (1982)	Mixed	38	39.5	40	35.0
Scotland Dumfriesshire Perchall Moss	NY11-87-	Shiny (1979)	Shiny	19	0	25	0
Scotland Dumfriesshire Kinmont	NY13-69-	Shiny (1981)	Mixed	4	0	2	100
Scotland Dumfriesshire Lochwood	NY08-96-	Shiny (1989)	Shiny	12	0	19	0
Scotland Roxborough Branxsholme Wester Loch	NT42-11-	Shiny (1975)	Mixed	18	33.3	16	12.5
Scotland Midlothian Whiteside Law	NT35-50-	Shiny (1988)	Mixed	19	10.5	34	20.6
Scotland Peebleshire Meldons-Eddleston	NT21-41-	Shiny (1982)	Shiny	18	0	29	0
Scotland Peebleshire Mount Bog	NT10-41-	Shiny (1979)	Shiny	17	0	26	0

## Materials and Methods

### Population status and temporal change

During 2007--2008, samples of *Hydroporus memnonius* were obtained from 27 populations in northern England and southern Scotland, spanning the transition zone between *castaneus* and shining female populations. Localities were those for which previous data on female status were available for the period 1974--1989 from the UK national water beetle recording scheme, administered by GNF, and included all sites from which mixed female populations had been previously reported (see Table 1). During both periods each locality was sampled for approx. 1 h, or until at ca. 20 *H. memnonius* adults were obtained, whichever occurred first. In all cases beetles were collected using a D-framed pond net (30 × 25 cm; 1 mm mesh), killed with ethyl acetate vapour, and preserved in 70% ethanol until examination. Individuals were sexed either by dissection of genitalia, or through the presence/absence of modified, enlarged sucker hairs on the underside of fore and mid tarsi (see Fig. 1 and Bilton, Thompson & Foster, 2008). In the 1970s/1980s populations were scored as shining, *castaneus* or mixed on the basis of female sculpture (differences between males were not appreciated at the time), using a variety of binocular microscopes. In the 2007--2008 survey, samples were screened using a Leica MZ8 stereomicroscope, at a magnification of ×20--×100, and the number of individuals of each form counted. Females were assigned to shining form or *castaneus* on surface sculpture; males were assigned to + or − form on the basis of the two large suckers present centrally on the second tarsomere of the fore and middle legs of + males (Fig. 1; Bilton, Thompson & Foster, 2008). Specimens are retained in DTB's collection (Plymouth), which will eventually be deposited in the Oxford University Museum of Natural History.

Differences in the frequency of shining, *castaneus* and mixed populations between 1974--1989 and 2007--2008 were analysed using a *G*-test in StatView 5.0.1. Whether there was a relationship between the proportion of + males and *castaneus* females in a population was assessed using a Spearman's correlation in SPSS 17.0.

### Assessment of thermal tolerance and acclimatory ability

#### Specimen collection, maintenance and experiment preparation

To assess possible differences in the thermal physiology of the *castaneus* and shining female forms of *H. memnonius*, and whether these are related to changes in their distributions, specimens were collected from Loch Doon, Ayrshire (shining) and Moorthwaite Moss, Cumbria (*castaneus*), as described above. Localities were chosen to be close to the contact zone between morphs, but consist entirely of either shining or *castaneus* individuals. After collection, beetles were transported to the laboratory in 1-L plastic containers filled with damp aquatic vegetation, kept within insulated bags (Thermos^®^, Rolling Meadows, IL, USA) in order to minimize thermal variation. In the laboratory, specimens were maintained in 5-L aquaria (max. 20 individuals per tank) containing aerated artificial pond water (pH 7.5; ASTM, 1980) in a 12:12 h LD regime, and fed live chironomid larvae *ad libitum*. Each population was divided into two equal groups, acclimated at 10 and 20 °C respectively for 7 days (Hoffmann & Watson, 1993; Klok & Chown, 2003; Terblanche & Chown, 2006; Calosi, Bilton & Spicer, 2008; Calosi et al., 2008). Temperatures were chosen as representative of fluctuations which will occur within typical *memnonius* habitats during periods of adult activity between spring and autumn. After acclimation, individuals from each of the two treatments were further randomly assigned to two equal subgroups: one used to measure upper thermal limits (UTL) and the other lower thermal limits (LTL). Finally, individuals were weighed (to ±0.001 g) using a Sartorius 1204 MP2 balance (Sartorius Ltd, Epsom, UK).

### Experimental procedure

Experiments to determine upper and lower thermal limits commenced at the temperature at which individuals of a given sub-group had been acclimated. A total of 60 individuals were used from each population, 15 for each acclimation temperature/thermal limit combination. Tests were carried out in air, employing a dynamic method and using a ramping program (±1 °C min), with a computer-controlled water bath (Grant LTC 6--30, using the Grant Coolwise software; Grant Instruments (Cambridge) Ltd, Herts, UK). Experimental ramping rate and equilibration temperature can influence the outcome of thermal tolerance tests (Terblanche et al., 2007; Chown et al., 2009), and consequently selecting ecologically realistic ramping rates is difficult when comprehensive environmental data are lacking. Therefore an identical ramping rate was employed, to allow comparisons amongst treatments and taxa (see Lutterschmidt & Hutchison, 1997a; Lutterschmidt & Hutchison, 1997b; Calosi, Bilton & Spicer, 2008; Calosi et al., 2008). Individuals were introduced, one per well (diam. 12 mm, depth 18 mm), into a generic 24-well plastic culture plate (Corning Ltd, Sunderland, UK), whose external base was painted white with Tipp-Ex^®^ to allow easy visualization of temperature-related responses. A maximum of 10 individuals were tested at any one time, and two investigators worked together, to ensure accuracy whilst recording thermal limits. Holes were made in the sides and bottom of the plates (but not the actual wells) to allow for maximum circulation of water (used for UTL experiments) or 70% ethylene glycol solution (used for LTL experiments) around the wells. The actual temperature within each well was measured directly using a calibrated digital thermometer (Omega^®^ HH11; Omega Engineering Inc., Stamford, CT, USA) equipped with an Omega^®^ precision fine-wire thermocouple (type K - dia./ga. 0.010 Teflon). Individuals were removed from their acclimation aquaria, quickly and carefully dried on absorbent paper, and placed into a clean, dry well. To avoid escape, plates were covered with a lid between additions of individuals. Once the experiment started, the lid was removed to avoid the build-up of water vapour, which can affect thermal tolerance.

### Determination of upper and lower thermal tolerance

Whilst a number of potential end-points were identified for tolerance to both heat and cold in preliminary experiments, lethal points were more repeatedly identifiable for both UTL and LTL as already documented for other diving beetle species (Calosi, Bilton & Spicer, 2008; Calosi et al., 2008). Death was readily identifiable in upper thermal tolerance experiments, as the point at which all movement of antennae and palpi ceased. Such cessation of movement was preceded by spasmodic movement of legs, antennae and palpi (Chown & Terblanche, 2007; Hazell et al., 2010). Beetles never revived following the cessation of movement in UTL experiments. Defining lower lethal limits proved more difficult than for upper ones, since individuals exhibiting total paralysis (or chill coma) would revive and recover full or partial locomotory abilities shortly after the end of the exposure period. As a consequence, paralysed individuals were instantly warmed to 30 °C for 3 s, and returned to the experiment if they recovered. This operation was first performed at 2 °C below the temperature at which total paralysis was initially observed, and was then repeated at regular intervals of 2 °C of cooling, until they no longer recovered and death was recorded (following Calosi et al., 2008).

### Data analysis

Data met assumptions of normality for both UTL and LTL (Shapiro--Wilks test), but did not meet assumptions for homoscedasticity of variance. Despite this, since there were four treatments with 120 individuals in total, an analysis of variance approach was employed (see Underwood, 1997). The effect of population, acclimation temperature and sex on UTL and LTL were examined using ANOVAs or ANCOVAs (with body mass as a covariate). When no significant effect of body mass and/or sex was detected these factors were dropped from models. All statistical analyses were conducted using SPSS v. 17.0.

## Results

### Population status and temporal change

**Figure 2:**
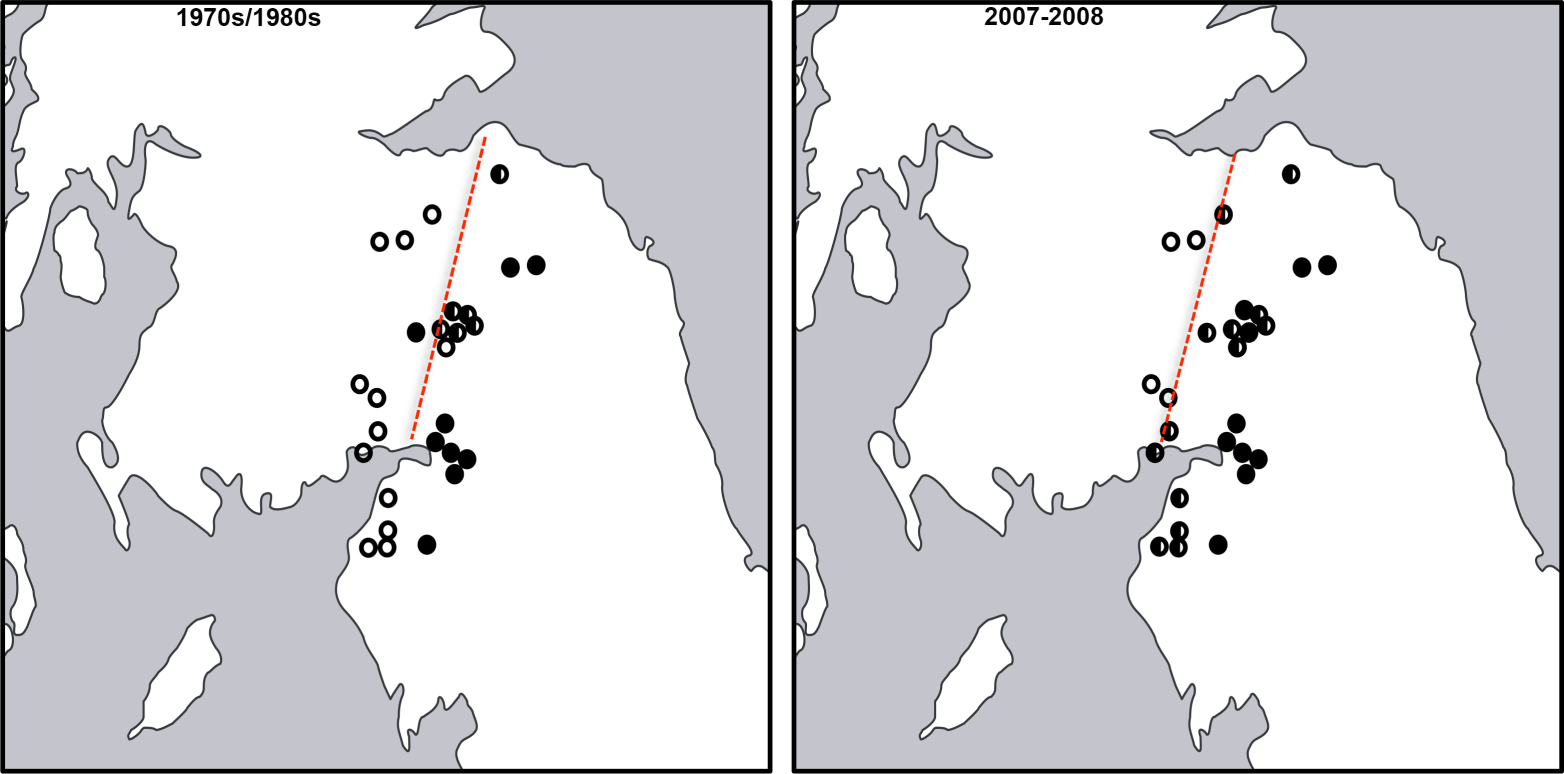
Map of *Hydroporus memnonius* contact zone. Status of studied populations of *Hydroporus memnonius* in northern England and southern Scotland in the 1970s/1980s and 2007--2008. White circles indicate shining female populations, black circles *castaneus*, white/black circles populations where both forms co-occur(ed). Red dotted lines indicate approximate position of the `leading edge' of *castaneus* on both occasions (see text).

**Figure 3:**
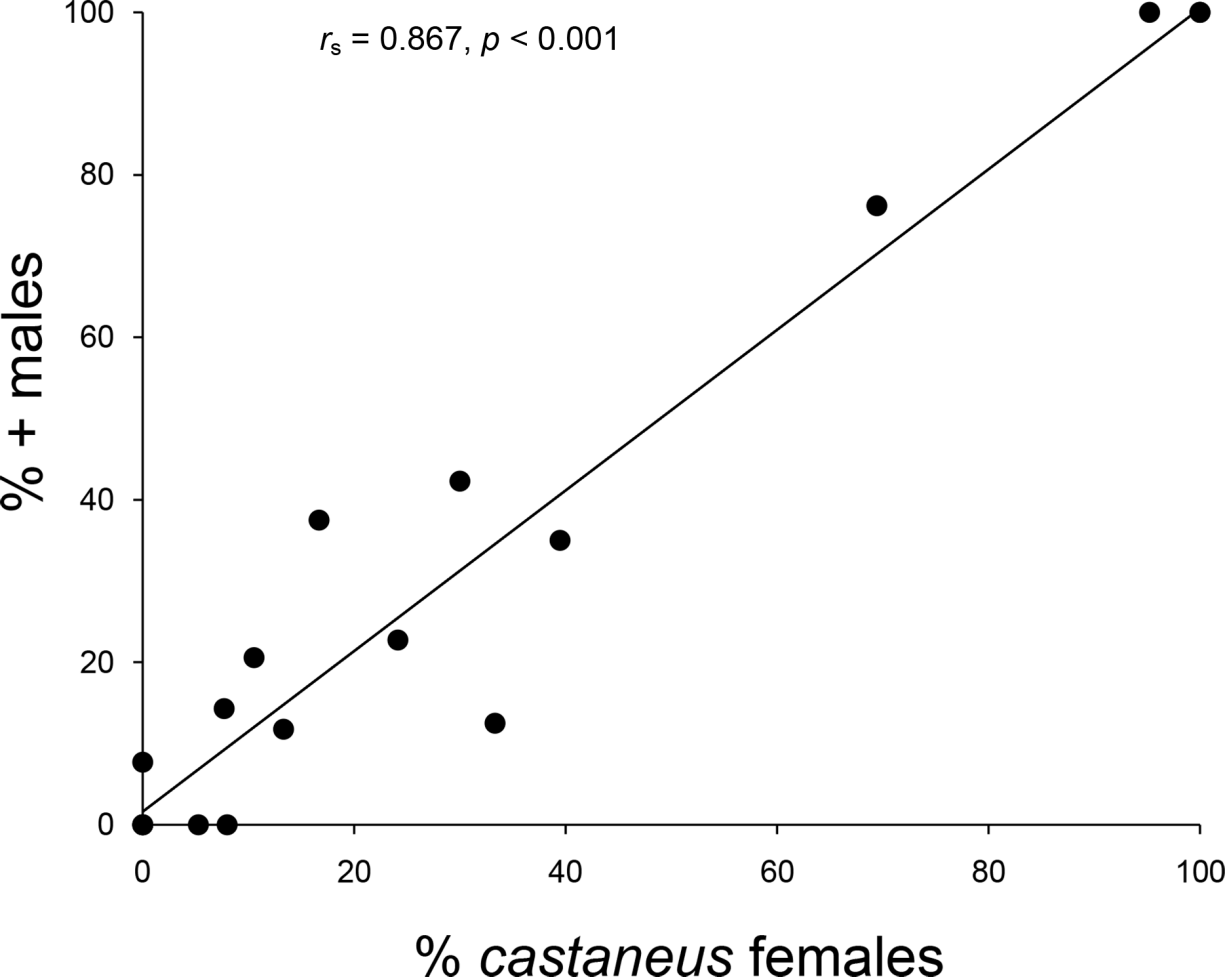
Relationship between male and female frequencies. Relationship between the percentage of *castaneus* females and + males in *Hydroporus memnonius* populations in northern England and southern Scotland. Note that points for 0 and 100% represent data from multiple populations.

The 2007--2008 population survey revealed that an expansion of *castaneus* females and associated + males had taken place across northern England and Southern Scotland since the 1970s/1980s (*G*_2_ = 6.376, *p* = 0.045). As can be seen from Table 1 and Fig. 2, eight populations composed of only shining females in 1970--1980 contained both forms by 2007--2008, including all four shining female *memnonius* populations previously reported in West Cumbria. Over the same time period, two of the mixed populations reported from the Scottish Borders shifted to being composed entirely of *castaneus* females and + males (Table 1; Fig. 2). The four other Borders populations reported as having both forms of female in the 1970s/1980s remained mixed in 2007--2008, and only a single case of an apparent expansion of the shining form was observed, in Riskinhope Moss, where a single shining female was found in 2008, in a population apparently composed entirely of matt females in 1985 (see Table 1). In 2007--2008 the percentage of + males in individual localities showed a strong positive correlation with the proportion of *castaneus* females (*r*_*s*_ = 0.867, *p* < 0.001 ---see Fig. 3).

### Thermal tolerance and acclimatory ability

The upper thermal tolerance of the shining female form was slightly but significantly higher than that of *castaneus* (ANCOVA *F*_1,59_ = 7.89, *p* = 0.007); the mean UTL of the shining form being 41.65 °C (±0.85 SD) compared to 41.29 °C (±0.83 SD) for *castaneus* individuals. Both populations showed a slight but significant positive acclimation of UTL in response to prior maintenance at higher temperatures (ANCOVA *F*_1,59_ = 10.82, *p* = 0.002); mean UTL being 41.76 °C (±0.8 SD), following 20 °C acclimation, and 41.18 °C (±0.81 SD) following acclimation at 10 °C. Females were significantly more tolerant of higher temperatures (ANCOVA *F*_1,59_ = 12.61, *p* = 0.001), their mean UTL being 41.77 °C (±0.96 SD) versus 41.23 °C (±0.68 SD) for males. Finally, body mass had a significant effect on UTL (ANCOVA *F*_1,59_ = 10.48, *p* = 0.002); larger individuals having higher UTLs.

In the case of lower thermal tolerance, *castaneus* had a greater cold tolerance than the shining form (ANOVA *F*_1,59_ = 4.90, *p* = 0.031), with a mean LTL of --8.69 °C (±1.92 SD) versus --7.73 °C (±1.58 SD). Populations also differed significantly in their response to prior acclimation temperature (ANOVA *F*_1,59_ = 6.05, *p* = 0.017). Individuals of *castaneus* showed a positive acclimation following exposure to lower temperatures, having a mean LTL of --9.49 °C (±1.65 SD) after maintenance at 10 °C, and --7.88 °C (±1.86 SD) after being kept at 20 °C. Beetles from the shining female population in contrast had a higher mean LTL after being cultured at 10 °C (--7.47 ± 1.68 SD) than at 20 °C (--7.99 ± 1.48 SD).

## Discussion

Whilst the contact zone between the two forms of *Hydroporus memnonius* remains situated in the same broad region close to the border between England and Scotland, our study reveals that *castaneus* has expanded at the expense of shining female form over the past 30 years. In all but one of the 27 populations investigated here there has been either a shift towards matt females and associated males, or no change during the study period. Matt females and + males were found in eight populations which contained only shining females in the 1970s and 1980s. This phenomenon has affected populations in both West Cumbria and the Scottish Borders, including all those closest to areas occupied by *castaneus* populations alone. On the basis of these data, the `leading edge' of *castaneus* penetration into Scotland, as estimated by the most north-westerly known mixed populations, has shifted approximately 40--50 km between the 1970s/1980s and 2007--2008. Whilst 30 years ago this was situated around a line running from Gretna in the southwest, to Dunbar in the northeast, it has now moved to a position roughly between Dumfries and Edinburgh (Fig. 2).

Only a single case of an apparent population shift in favour of the shining female form was detected, in Riskinhope Moss, Selkirkshire. Here a population which was apparently composed entirely of *castaneus* in 1985 was scored as mixed in 2008, on the basis of the presence of a single shining female amongst 54 scored individuals. The occurrence of the shining female form at such a low frequency may have resulted from the dispersal of individuals from surrounding shining or mixed populations, since Riskinhope Moss is close to the leading edge of *castaneus* penetration into Scotland, meaning that such populations are likely to remain undetected in the region. *Hydroporus memnonius* is known to fly (Bilton, 2014), and can colonize suitable habitats relatively rapidly, including new ponds (DT Bilton, pers. obs., 1983), meaning that dispersal of individuals could account for such specimens. Alternatively, the Riskinhope Moss population may have been mis-scored in the 1980s; rare shining females being overlooked amongst males. Whilst the populations scored in the 1970s and 1980s were examined systematically for the two female forms, the differences between + and − males were not appreciated at the time, and it is possible that in a few cases shining females were passed off as males, which they resemble very closely (Fig. 1; Bilton, Thompson & Foster, 2008). However, it is worth noting that any possible mis-scoring does not detract from the finding of *castaneus* expansion. If anything, the distinctiveness of the matt form is likely to have caused it to be over-reported in the past, making its north-westerly expansion of 40--50 km all the more striking.

A number of recent studies have demonstrated a strong link between temperature tolerance and both geographical range size and the latitude of the range centre in dytiscids, thermal physiology typically being the best predictor of these parameters (e.g., Calosi, Bilton & Spicer, 2008; Calosi et al., 2010). In the case of *H. memnonius* however, differences in thermal physiology between the two populations examined, whilst significant, are relatively slight, and seem unlikely to be involved in the observed northward and westward expansion of *castanaeus*. Whilst our data are restricted to single populations of each morph, such an approach been shown to be sufficient to characterise biogeographically meaningful differences in thermal physiology between other closely related dytiscid species (e.g., Calosi, Bilton & Spicer, 2008; Calosi et al., 2010). Here the *castaneus* form appears to have lower heat tolerance and higher cold tolerance than the shining, meaning that an *increase* in the occurrence of the shining form may have been predicted in the contact zone over the study period, if distributional changes were driven by climate warming. As stated above, the global distribution of the two forms is also highly unlikely to be driven by consistent physiological differences between them, as shining populations are essentially peripheral to *castaneus*, being found in areas of relatively warm climate around the Mediterranean and also in the west and north, including Scandinavia, where they experience much lower temperatures. Additionally, as noted by Bilton, Thompson & Foster (2008), available mitochondrial DNA data for *H. memnonius* show that the two forms are not reciprocally monophyletic and suggest that *castaneus* and its associated + male have become fixed in a number of different genetic lineages.

Instead of changes in temperature, the observed expansion of the matt female form of this water beetle may be driven by the dynamics of sexual conflict. Härdling & Karlsson (2009) model the outcomes of sexually antagonistic coevolution with dimorphic males and females and phenotypic matching, whereby the mating probability in encounters between heterotypic individuals is lower than that between homotypic ones. They show that dimorphisms may persist in populations due to negative frequency dependent selection, although this outcome is sensitive to differences in mating system. Although temporal comparisons have not been made to date, a stable persistence scenario may hold for some dytiscid systems (e.g., *Graphoderus zonatus* (Hoppe); Bergsten, Töyrä & Nilsson, 2001), where male adaptations associated with rough female resistance traits impair their ability to attach to smooth females and vice versa (Karlsson Green et al., 2013). The situation in *H. memnonius* seems to differ from those modelled by Härdling & Karlsson (2009), however, as our study demonstrates and populations in which there is a mixture of the two forms are both rare and transient. Specifically, the reciprocal trade-offs modelled and observed in some diving beetles do not appear to apply to this *Hydroporus*, where + males would be expected to be more able to overcome resistance in mating encounters with either female morph, due to their greater ability to attach to both rough and smooth surfaces (Bilton, Thompson & Foster, 2008). In such a situation, *castaneus* and its associated + male would be expected to replace the shining form where the two come into contact --precisely what we observe over the 20--30 year span of our study. Indeed once the matt form and associated male invade a shining female population their characteristics may achieve fixation relatively rapidly, the strong correlation between the frequency of male and female morphs observed in mixed populations being suggestive of strong linkage disequilibrium between resistance and persistence traits. As *castaneus* comes into contact with the shining form, alleles coding for persistence traits of + males (wider tarsi, differences in articulo-setae) would be expected to increase in frequency as they would result in greater mating success on either female morph. As the frequency of + males increases in a population, shining females would be expected to experience increasingly lower fitness, due to higher mating rates, something which is better resisted by matt females, whose frequency would instead be expected to increase. In a similar manner, − males would be at an increasing disadvantage as matt female characters invade a population, due to their more limited attachment ability. Over time, these processes could lead to the replacement of shining female populations by *castaneus*, the speed at which this occurs likely being determined by local population density and the rate of supply of individuals of the two morphs.

## Conclusion

Using data collected over a thirty year period, we show that there has been a significant expansion of the matt *castaneus* female form of *Hydroporus memnonius* where it meets the shining, male-like form. Matt females and associated + males have replaced shining females and − males throughout the contact zone in northern England and southern Scotland. On the basis of both thermal tolerance data and the wider global distribution of these morphs we argue that the observed range shift is highly unlikely to have resulted in response to climate warming, but is instead consistent with predictions based on the dynamics of sexually antagonistic coevolution in this beetle. Future work on this system, particularly studies exploring relative mating success in heterotypic and homotypic encounters would prove most illuminating.

##  Supplemental Information

10.7717/peerj.2089/supp-1 Supplemental Information 1 *Hydroporus memnonius* thermal tolerance data

## Additional Information and Declarations

### Data Availabilty

The following information was supplied regarding data availability: The raw data has been supplied as Supplemental Information.
